# DNA Released by Adeno-Associated Virus Strongly Alters Capsid Aggregation Kinetics in a Physiological Solution

**DOI:** 10.1021/acs.biomac.4c00027

**Published:** 2024-04-29

**Authors:** Curtis W. Jarand, Karen Baker, Matthew Petroff, Mi Jin, Wayne F. Reed

**Affiliations:** † Department of Physics, Tulane University, New Orleans, Louisiana 70118, United States; ‡ Downstream and Drug Product Process Development, Spark Therapeutics, Philadelphia, Pennsylvania 19143, United States

## Abstract

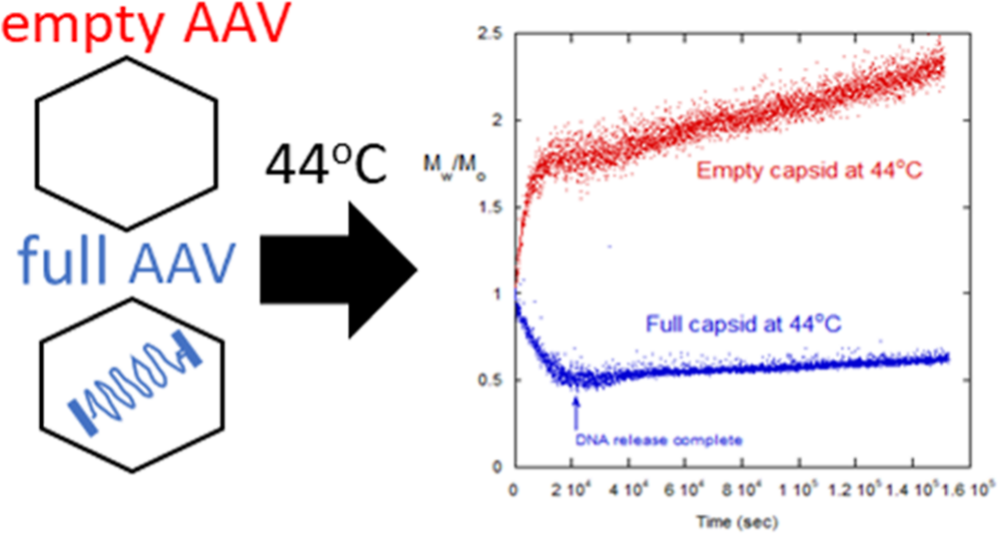

While adeno-associated virus is a leading vector for gene therapy, significant gaps remain in understanding AAV degradation and stability. In this work, we study the degradation of an engineered AAV serotype at physiological pH and ionic strength. Viral particles of varying fractions of encapsulated DNA were incubated between 30 and 60 °C, with changes in molecular weight measured by changes in total light scattering intensity at 90° over time. Mostly full vectors demonstrated a rapid decrease in molecular weight corresponding to the release of capsid DNA, followed by slow aggregation. In contrast, empty vectors demonstrated immediate, rapid colloid-type aggregation. Mixtures of full and empty capsids showed a pronounced decrease in initial aggregation that cannot be explained by a linear superposition of empty and full degradation scattering signatures, indicating interactions between capsids and ejected DNA that influenced aggregation mechanisms. This demonstrates key interactions between AAV capsids and their cargo that influence capsid degradation, aggregation, and DNA release mechanisms in a physiological solution.

## Introduction

Rare diseases are an increasingly significant healthcare burden, of which approximately 80% are due to single-gene genetic disorders.^1−3^ Recombinant adeno-associated virus (rAAV) is a leading vector for the treatment of single-gene disorders, with over 100 active clinical trials worldwide.^4^ AAV is a nonenveloped virus, with an approximately 25 nm diameter protein capsid that can deliver a single-stranded DNA payload of typically 4.5 kilobases.^5,6^ Advantages of rAAV over other gene therapy vectors include high transduction efficiency, low immunogenicity relative to other viral vectors, and varied tropism across AAV serotypes enabling preferential delivery of cargo to various tissues.^5−7^

One active area in AAV research is understanding the viral vector degradation process, i.e., the pathways of physical and chemical change that may impact infectivity, transduction, and immunogenicity. For a viral vector, these may include capsid disruption and cargo release^8,9^ in addition to common protein routes such as fragmentation, aggregation,^10^ and oxidation.^11^ Such insights are critical to drug development^11^ including (a) designing appropriate storage strategies for AAV drug product,^12−14^ which are currently heavily reliant on cumbersome ≤−60 °C cold chains,^15^ (b) design of analytical methods to characterize key impurities that form during storage,^11^ and (c) fundamental understanding of the gene transduction process.^16,17^

Significant efforts to understand AAV stability have been made using thermal shift assays^18−22^ to probe elevated temperatures at which capsids expose or release cargo (e.g., uncoating) and their constituent capsid proteins unfold. These studies generally report AAV uncoating at around 50–60 °C and viral protein unfolding at around 70–90 °C. While insightful, these are somewhat removed from the much lower temperatures of viral transduction (37 °C), viral production (25 °C), and pharmaceutical storage (−80 to 8 °C). Equally important is that the stability of the AAV structure measured from these assays does not incorporate potentially destabilizing colloidal and interfacial effects.

Fewer efforts have been made to study the viral vector degradation process. In two excellent studies, Horowitz et al.^22^ and Bernaud et al.^9^ used single-particle techniques to study the effects of short-term heat stress on several AAV serotypes. For increasing stress temperatures and duration, they reported a general transition from initially intact vectors to seemingly intact vectors with externalized DNA and eventually to completely ruptured capsids and/or capsid fragments with aggregated DNA. However, these used short-term stresses at very high temperatures (50–80 °C) that were generally above the AAV uncoating temperature. As such, they are predominately measuring degradation due to large structural changes and are highly abstracted from the degradation and uncoating processes of cellular transduction or pharmaceutical storage which occur below the temperature for rapid uncoating.

Studies of AAV degradation at lower temperature have focused on the effects of long-term storage and drug product formulation strategy on vector infectious titer and/or aggregation.^12,14^ While directly studying conditions relevant toward pharmaceutical storage, AAV material scarcity limited these studies to a sparse set of time points, providing limited kinetic insight. In addition, these studies have not assessed the effects of DNA payload or product variants such as particles without DNA genome (“empty” viral particles) on drug product stability. This is a notable gap, as empty vector and DNA transgene are not only present in the AAV drug product but also likely degradation byproducts^9,23^ and thus may impact a drug product’s shelf life.

One alternative to study macromolecule degradation is the use of time-resolved total intensity light scattering. This has been widely used for proteins^24−26^ and inorganic colloids^27^ and uses time-dependent changes in the total intensity of scattered light to measure changes in the average molecular weight of particles in a solution. While an ensemble-average measurement cannot distinguish between multiple simultaneous species, its continuous measurements provide high-temporal resolution of kinetics. Studies are also compatible with moderate temperatures (25–60 °C) that can study degradation below the uncoating temperature and thus are less abstracted from physiological, processing, and storage temperatures. To date, such methods have chiefly been used to study protein solutions^26,28^ and early phase colloidal aggregation.^27^

This work studies the degradation of full and empty AAV vectors to understand the degradation kinetics and mechanisms. Total intensity light scattering was used to monitor molecular weight changes of a proprietary engineered AAV. Mostly full AAV was exposed to temperatures of 30–60 °C for up to several days. Degradation of mostly full AAV resulted in an early molecular weight decrease. In contrast, empty vectors exhibit a significant, immediate, and large-scale increase in aggregation rate. Studies probing payload ratio and addition of nuclease to degrade released DNA show that the difference between aggregation of empty vs full capsids is due to the presence of ejected DNA cargo and associated proteins that then interact with the disrupted and/or empty viral capsids. This provides an important contribution to the understanding of AAV uncoating and degradation processes that will be useful in research on vector transduction and drug product storage.

The results also pose a fundamental question about the mechanism by which DNA release dramatically slows the aggregation of the empty capsids. A mechanism is conjectured below, but there is a need for a better theoretical framework for understanding these phenomena. While a difference in capsid protein type or conformation between empty and full capsids cannot be excluded as part of an explanation of the mechanism, the fact that the presence of a relatively small amount of capsids whose DNA has been released should influence the capsids of different protein embodiments does not seem to support this interpretation.

## Materials and Methods

### AAV Capsids and Solutions

This study used a Clade E engineered AAV capsid with a 4.7 kilobase ssDNA genome, produced with standard methods as follows, and similar to that described previously.^29^ AAV vectors were produced using triple transfection of HEK-293 in suspension cell culture, lysed with Triton-X100, and clarified with centrifugation and 0.2 μm filtration. AAV was purified from cell lysate by using affinity chromatography (AAVX capture select). Capsids with vector genome (“full”) and without vector genome (“empty”) were obtained from anion-exchange chromatography fractions enriched in empty capsids (low-salt concentration chromatography wash) and full capsids (high-salt concentration chromatography elution). Purified empty and full capsids were further concentrated and buffer-exchanged into a 20 mM sodium phosphate and 180 mM NaCl, pH 7.3 solution. Endonuclease (Benzonase, Sigma-Aldrich, CAS 9025-65-4) was used to degrade DNA in solution. Capsids, benzonase, and buffer solutions were filtered before use using a 0.2 μm filter.

AAV samples were characterized for a concentration of capsids with encapsulated viral genome (vg, “full”) and total capsids (cp, “full” + “empty”) and using size-exclusion chromatography with multiangle light scattering (SEC-MALS; Sepax SRT Sec-500, Sepax Technologies; Dawn Helios, Wyatt) as described previously.^30^ Relative changes in total DNA and encapsulated vg were detected using quantitative polymerase chain reaction (qPCR) as described^31^ and run with (vg) or without (total DNA) predigestion with nuclease to degrade soluble DNA. Relative changes in empty and full capsid concentration were also monitored using analytical anion-exchange chromatography (HP-AEX) eluted with a NaCl concentration gradient at pH 9.

### Static Light Scattering and Dynamic Light Scattering

Time-dependent static light scattering (SLS) measurements were performed with ARGEN (Fluence Analytics) and dynamic light scattering (DLS) (NanoBrook Omni, Brookhaven Instruments). ARGEN consists of 16 independent 90° SLS compartments, each with its own laser, vertically polarized beam of 660 nm wavelength, temperature control, and stepper-motor controlled stir rate. It allows 16 measurements of scattering intensity to be made simultaneously, and individual samples can be inserted and removed at any time without affecting other ongoing measurements. The general name for the parallel sample method is simultaneous multiple sample light scattering (SMSLS).^32^

#### DLS Analysis^32^

For a spherical particle in a solvent of viscosity, η, and single hydrodynamic diameter, *d*_H_, the usual Stokes–Einstein equation is used to relate *D*_0_, the particle’s self-diffusion coefficient, and *d*_H_, the hydrodynamic diameter^33^
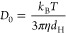
1where *k*_B_ is Boltzmann’s constant. The *z*-average self-diffusion coefficient, ⟨*D*_0_⟩_*z*_, is related to the *z*-averaged reciprocal hydrodynamic diameter ⟨*d*_H_⟩_*z*_ by
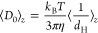
2so that the “hydrodynamic diameter” reported by commercial DLS instruments (including the Brookhaven DLS instrument) is only an “apparent hydrodynamic diameter”, ⟨*d*_H_⟩_*z*,ap_, which is the reciprocal of the *z*-average reciprocal hydrodynamic diameter^33^
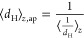
3

⟨*d*_H_⟩_*z*,ap_ is equal to the true *z*-average hydrodynamic diameter <d_H_>_z_ only for monodisperse populations of spheres. While small particles (Rayleigh scatterers) scatter linearly polarized light equally in all directions in the plane perpendicular to the polarization direction, as particle size increases, there is less scattering at higher angles (0° is in the direction of the incident laser beam and 180° is the full backscatter direction), so that at finite angles, larger particles will be under-counted and ⟨*d*_H_⟩_*z*,ap_ will underestimate the size of the particles the higher the angle.^34^ These effects are discussed below in the evaluation of the SLS and DLS results.

The polydispersity index, PI, can be obtained from DLS data when the logarithm of the scattered intensity autocorrelation function is expanded as a second-order polynomial in correlation time. The ratio of the second-order coefficient to the square of the first-order coefficient is the PI and is a rough measure of the width of the particle distribution. A perfectly monodisperse population has PI = 0, and PI < 0.10 is usually considered a fairly narrow distribution, whereas PI > 0.3 is considered highly polydisperse.

##### SLS Analysis^34^

Analysis started with the usual dilute solution expression for determining weight-average molar mass *M*_w_(35)
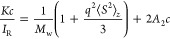
4where *c* is the mass concentration (g/cm^3^) of scatterers, ⟨*S*^2^⟩_*z*_ is the *z*-average mean-squared radius of gyration, *A*_2_ is an averaged second virial coefficient, *I*_R_ is the measured absolute Rayleigh ratio (determined by reference to *I*_R_ = 1.183 × 10^–5^ cm^–1^ for toluene at 660 nm and *T* = 25 °C), and *q* is the magnitude of the scattering vector^35^
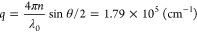
5Here, *n* = 1.33 is the index of refraction of the aqueous solvent, λ_0_ = 660 nm is the vacuum wavelength of the incident laser, and θ is the scattering angle, 90°. *K* is an optical constant, which for linearly polarized light with the electric field perpendicular to the horizontal scattering plane is^35^
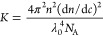
6where *N*_A_ is Avogadro’s number and d*n*/d*c* is the differential refractive index increment of the scatterer in its solution, taken as 0.185 cm^3^/g. Most of the results below are expressed in terms of *M*_w_(*t*)/*M*_0_, where *M*_w_(*t*) is the weight-averaged molar mass of all capsids, intact and aggregated, and *M*_0_ is the molar mass of the unaggregated, intact capsid. As such, the d*n*/d*c* = 0.185 cm^3^/g is only used for absolute *M*_w_ determinations, which appear in Table 1and Figures 2d and 4d.

**Table 1:** Molar Masses of Full and Empty Capsids and DNA

particle	molar mass (g/mol)	measurement % error
empty capsid	3.4 × 10^6^	13.7%
full capsid	5.8 × 10^6^	12.5%
DNA (from sequence)	2.4 × 10^6^	NA

The fractional errors from use of single-angle detection at 90° are assessed below, as well as the neglect of the *A*_2_ term.

Table 1 shows the molar masses of empty and full capsids and DNA. Due to concentration uncertainties, the molar mass for full capsids was inferred by pooling release data on mixed empty and full capsids and finding the most self-consistent mass. Measured masses by SLS are all similar to the expected mass within a reasonable error, with the difference close to the expected mass of DNA from the sequence (2.2 vs 2.4 × 10^6^ g/mol).

##### Error Assessment Using 90° SLS

The multisample system used in this work has allowed 16 different experiments to run in parallel. The unit has only θ = 90° detection, so it is important to assess the approximations and error bounds. Determination of *M*_w_ at finite angle, as opposed to extrapolation to θ = 0°, is within the low-angle approximation, and ignoring second virial coefficient (*A*_2_) effects leads to an apparent molar mass *M*′
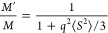
7

For empty and full capsids, for which *D*_H_ ∼ 22 nm, taken as uniformly dense spheres
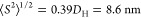
8

For *q* = 1.79 × 10^5^ (cm^–1^), this yields an underestimate of capsid *M* at θ = 90° detection of less than 0.1%, and so determinations of the unaggregated full and empty capsids can be taken as accurate.

**Figure 1:**
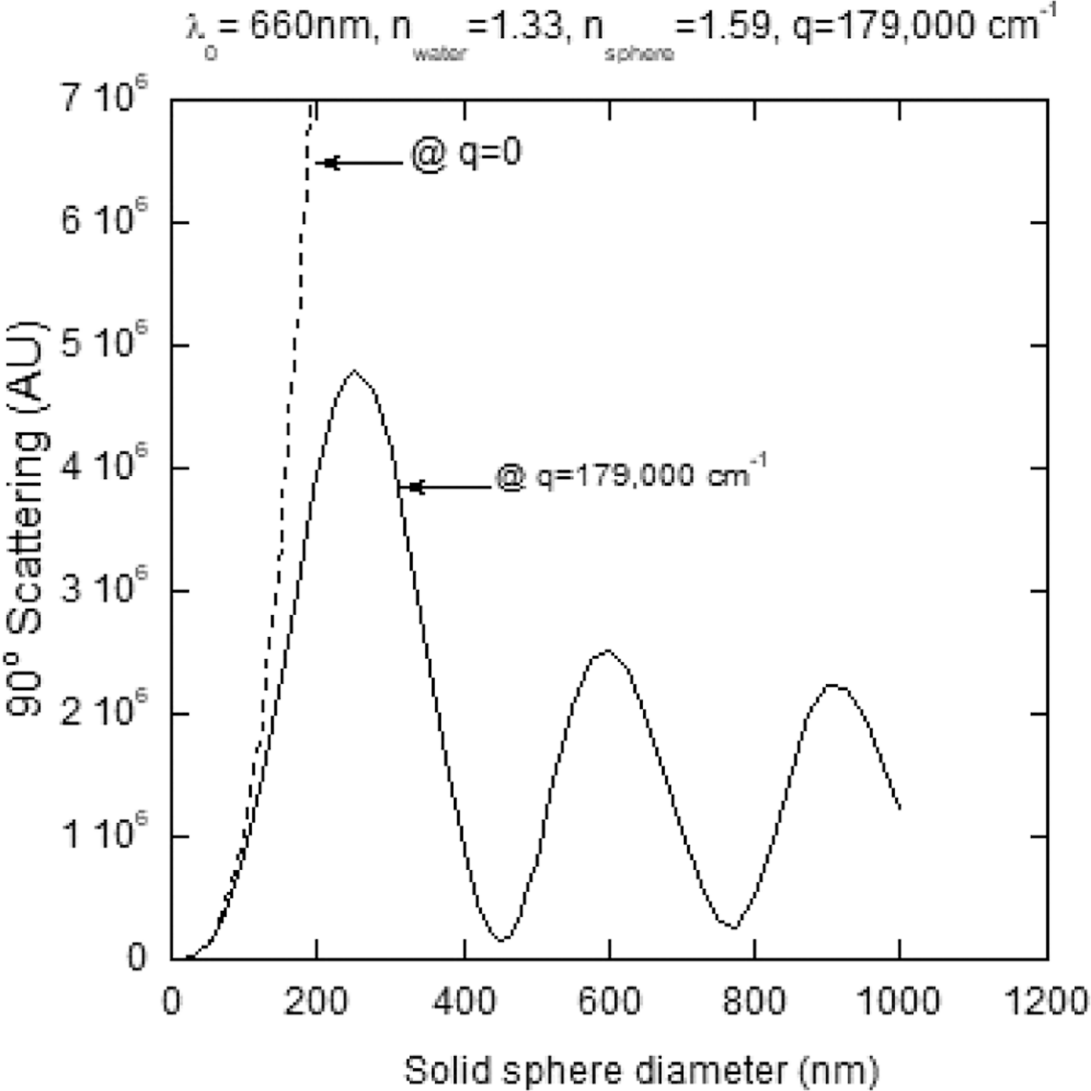
Intensity of scattering at 90° for *q* = 179,000 cm^–1^ as a function of sphere size. The dashed line represents the scattering at *q* = 0.

**Figure 2:**
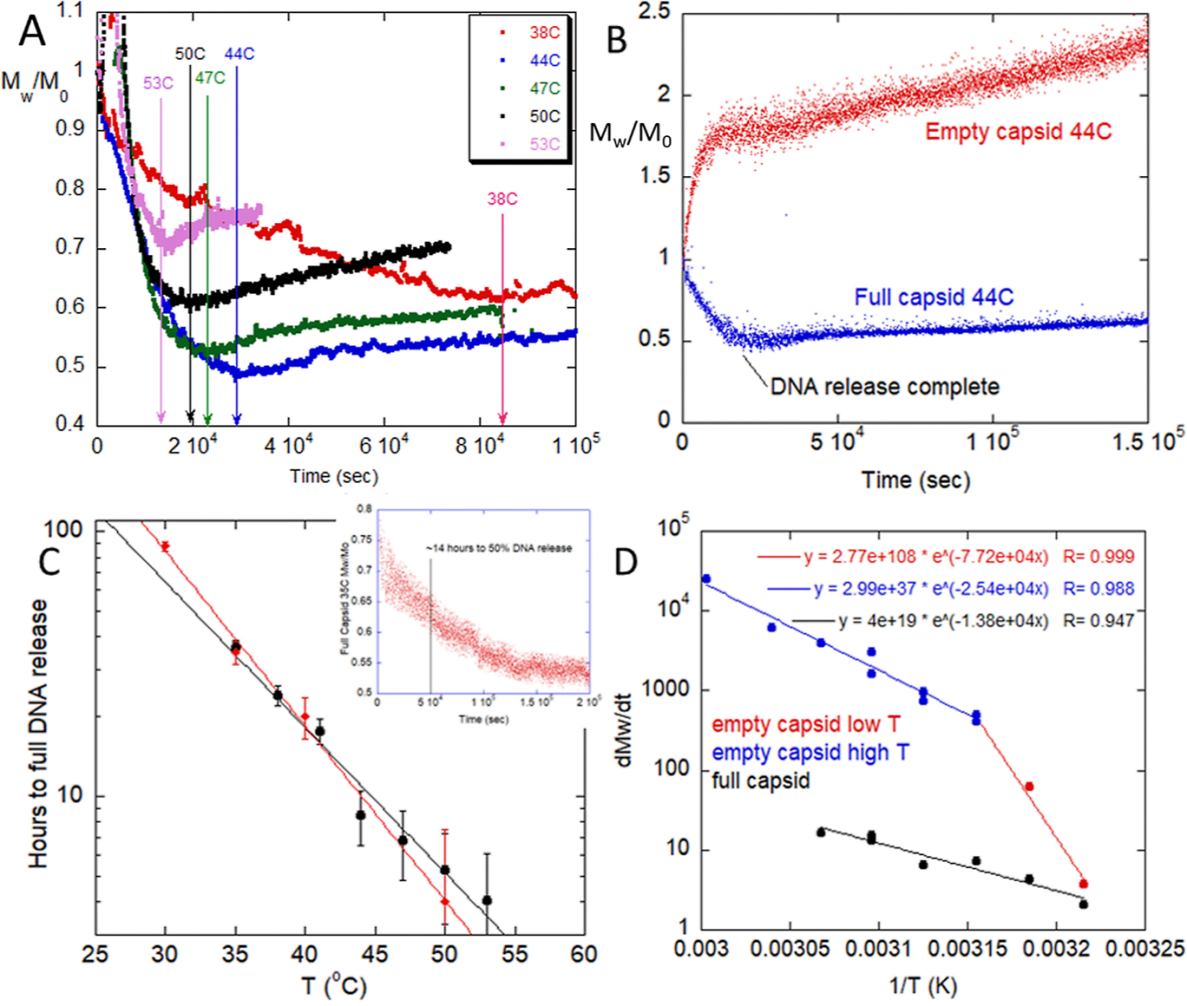
Full and empty AAV have distinct thermal degradation behavior. (A) Thermal degradation of 60% full AAV capsids from *T* = 38–53 °C. *M*_w_ is the weight-average molar mass of all scatterers at any time *t* > 0 and *M*_0_ is the molar mass of all scatterers at *t* = 0. *M*_w_ initially decreases over time, representing DNA release from the capsids, followed by slow aggregation. (B) Example thermal degradation of empty vs 60% full AAV at 44 °C. For full capsids, a characteristic decrease in *M*_w_ over time (DNA release from the capsids) is followed by slow aggregation. In contrast, the empty capsids begin with immediate aggregation. (C) Time to complete release of DNA estimated from (black) the SLS *M*_w_/*M*_0_ minimum and (red) titers of quenched AAV samples further analyzed by HP-IEX. Inset depicts profile at 50% DNA release. (D) Arrhenius-like plots for empty capsid aggregation and the rate of DNA release (measured here as the reciprocal of time to full DNA release).

The error in measuring *M* for DNA at scattering angle θ = 90° using ⟨*S*^2^⟩^1/2^ = 29 nm is a bit larger and gives an underestimate of mass *M*′/*M* = 0.09. While these finite-angle errors are small, the approximation begins to fail dramatically as the aggregates get large. Approximated as spherical aggregates, by an aggregate diameter of 100 nm, there are on the order of 100 aggregates involved. The Rayleigh–Debye approximation is a good first approximation for small aggregates. Mie scattering effects become prominent for the spherical aggregates as their size increases. Figure 1 shows how the intensity of scattered light varies at 90° as the sphere diameter and mass increase. The maxima and minima are typical of Mie scattering effects. The dashed line shows how scattering intensity increases as the sixth power of diameter at *q* = 0, which is where angular Mie effects are eliminated. For the Mie computations, the index of refraction of the particles was taken as *n*_p_ = 1.59 and for the solvent *n*_s_ = 1.33. The computations were carried out on an online Mie Calculator by Scott Prahl (Figure 1 is merely illustrative and using a slightly different value of *n*_p_ = 1.59 will not affect the trends shown. Note that this computation is unrelated to computations made in Table 1 and Figures 2d and 4d, for which d*n*/d*c* = 0.185 cm^3^/g).

It is emphasized that in this work, aggregation rates were determined very early in aggregation, before large Mie effects set in. Figure 1 shows that, for example, there is about a 40% underestimate of scattering intensity at 90° due to the Mie effect at 150 nm, which corresponds to roughly 350 aggregated capsids. The Halo particle sizing (described below) shows that there are particles 100 s of nm up to many microns. These scatter very little light at 90° and show their effect by making the width of the SLS band broader and “noisier”. The “noise” fluctuations are the effect of individual or small groups of large particles (>300 nm) passing through the scattering volume.

##### SLS Error Assessment Neglecting *A*_2_

For the capsids of diameter *d* = 22 nm, *A*_2_ can be estimated by the hard-sphere expression
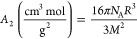
9where *R* is the radius of the sphere, taken from DLS as *R* = 1.1 × 10^–6^ cm, and *M* is the capsid mass, taken from Table 1, for the empty and full capsids. This gives
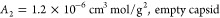
10a
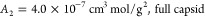
10b

With reference to eq 4, when the  term is negligible, as just shown for intact empty and full capsids, the ratio of the 2*A*_2_*c* two-body interaction term to the 1/*M* term is 2*A*_2_*Mc*, so that for the 2*A*_2_*c* term to be negligible, 2*A*_2_*Mc* ≪ 1.

For the concentration of empty capsids used, *c* = 2 × 10^–5^ g/cm^3^
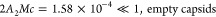
11aand for the concentration of full capsids used, *c* = 8 × 10^–6^ g/cm^3^
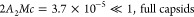
11b

This shows that steric interparticle interactions between full capsids and between empty capsids is entirely negligible at the concentrations used. The moderate level of ionic strength largely screens any electrostatic interactions.

##### Aggregation Rates from SLS

In addition to the rate of change of absolute molar mass, a useful dimensionless mass was also used to assess the kinetics, *M*_w_/*M*_0_, where *M*_0_ is the initial molar mass of the sample. The aggregation rate, AR (s^–1^), is taken as the initial linear slope of *M*_w_/*M*_0_ versus time
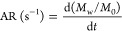
12

The interpretation of AR is the fractional increase in weight-average molar mass per second due to aggregation.

ARGEN sample preparation was performed by first filtering the buffer through a 0.22 μm syringe filter into a scintillation vial prior to sample preparation. The total sample volume for ARGEN analysis was 1 mL, and samples were prepared directly in 1 cm path length disposable polystyrene cuvettes before introduction to the preheated sample cells of the ARGEN instrument. Experiments for full capsid were prepared at a 50× dilution from stock samples, giving a working concentration of 0.008 mg/mL (1 × 10^12^ cP/mL). Empty capsid experiments were performed at a 40× dilution, giving a working concentration of 0.02 mg/mL (3 × 10^12^ cP/mL). Buffer and toluene background values were measured for each cell of the ARGEN instrument for *M*_w_–*M*_0_ and *M*_w_ calculations.

DLS samples were prepared in the same manner, but at 4× the working concentrations of the ARGEN samples to give an adequate count rate on the DLS. Whereas SLS provides *M*_w_, DLS yields a *z*-average diffusion coefficient ⟨*D*⟩_*z*_. At the very low concentrations of capsids used here, this diffusion coefficient is the *z*-averaged self-diffusion coefficient, ⟨*D*_0_⟩_*z*_, given in eq 2.

##### Particulate Analysis

Particulate analysis was performed using backgrounded membrane imaging, or BMI (Aura, Halo Laboratories).^36,37^ Background images were acquired of empty filter plate wells, sample was added and filtered to retain micrometer-sized particulates, membranes were rinsed and filtered with DI to remove soluble well residues, and then images were reacquired with both brightfield and darkfield illuminations. Images were analyzed for particulate size and morphology using the device software Particle Vu 3.2. Particles with a combination of circularity below 0.15 and side illumination (a dark-field analogue) intensity above 30 were extrinsic contaminants introduced during the BMI measurement (such as dust) and excluded from analysis. All manipulations were performed in a HEPA-filtered enclosure to reduce contamination from extrinsic particulates.

## Results

### AAV Thermal Degradation Kinetics Monitored by Time-Dependent SLS

AAV stocks under moderate thermal stress were monitored using SLS to understand the net molecular weight changes. Example SLS profiles at between 38 and 53 °C are shown in Figure 2A, for a 60% full AAV stock at approximately 1 × 10^12^ vg/mL in a 180 mM NaCl and pH 7.3. Data in Figure 2A were smoothed by a running window averaging for visual clarity. This plots the solution-average molecular weight (*M*_w_) normalized by the measured *M*_w_ at *t* = 0 (*M*_0_), used to find AR from eq 12.

In Figure 2A, thermal degradation of the mostly full AAV exhibits two main events: an initial decrease in molecular weight, followed by a much slower aggregation. Both events are strongly temperature dependent, with increasing rates at higher temperatures. Similar light scattering monitoring of the intensity decrease due to DNA release from bacteriophage T5 was reported earlier, using a membrane protein receptor to stimulate DNA release.^38^ The results also agree with single-molecule studies reporting initial DNA ejection followed by higher-order aggregation of disrupted capsids.^9,22^

Figure 2B compares the SLS profiles of a 60% full AAV to a 100% empty AAV at 44 °C. The degradation of empty AAV is in sharp contrast to the 60% full. The *M*_w_ for the empty AAV exhibits rapid, immediate aggregation, which slows after about 10,000 s (3 h) but continues to rise over the next 125,000 s (35 h). In contrast, the mostly full capsid solution decreases in *M*_w_ up to 25,000 s (7 h) before beginning a slow rise over the next 125,000 s (35 h). The aggregation of the empty AAV is notable in that, in addition to aggregating without a delay, its aggregation occurs much faster than that of the mostly full AAV during its later aggregation phase. These general degradation profiles—*M*_w_ decrease followed by slow aggregation for mostly full AAV and rapid *M*_w_ increase for empty AAV—were seen across the 35–60 °C temperature range of light scattering experiments in this study. This more than 100-fold difference in aggregation rates is not explainable by the less-than 3-fold capsid number density differences across empty and full samples and interfacial effects. It instead likely represents fundamental differences in the thermal degradation behavior of empty versus full AAV.

A notable difference between the SLS profiles is the initial decrease in *M*_w_ observed for the full but not empty AAV. To further understand this *M*_w_/*M*_0_ decrease, independently prepared full AAV solutions were sampled at various time points during thermal stress, rapidly quenched to 4 °C, and further characterized. Two orthogonal methods to measure encapsulated genome titer, HP-IEX and qPCR (further discussion in the Supporting Information), showed temperature-dependent titer decreases corresponding to the *M*_w_/*M*_0_ decrease observed from SLS in Figure 2A,B.

Estimates of the time to full genome release are plotted in Figure 2C. These vary between 4 and 40 h at 53–35 °C for both methods, suggesting that the main cause of the *M*_w_/*M*_0_ decrease is loss of the genetic cargo. Extrapolation to 60 °C predicts 0.9 and 1.4 h (quench vs SLS, respectively), which agrees with previous studies where AAV8 and AAV9 eject large fractions of their genome after 15 min exposure above 60 °C.^9,22^ Notably, these studies indicated further capsid degradation after additional thermal stress, which was observed to some degree by our HP-IEX assay, with the formation of uncharacterized degradant species.

The SLS signals during thermal stress (Figure 2A,B) are also further interpreted to determine temperature-dependent degradation rates and are shown as Arrhenius plots in Figure 2D. Notably, Arrhenius models have had limited success in extrapolating protein aggregation across wide temperature ranges.^10^ As such, we do not intend this for specific interpretation of low-temperature degradation rates and storage lifetimes but instead illustration of the general degradation behavior. Here, for the 60% full AAV, the time to reach the minimum *M*_w_/*M*_0_ was taken as the time for full DNA release. The reciprocal of this is effectively a rate and obeys the Arrhenius behavior seen in Figure 2D, with a low activation energy of around 25 kcal/mol. This activation energy is similar to a previous measurement for bacteriophage uncoating (28 kcal/mol)^38^ and quite low relative to aggregation of globular proteins which are typically 100–140 kcal/mol.^39^

A few factors may contribute to the relatively low activation energy of viral uncoating. First, DNA ejection likely does not require full disruption of the capsid, with multiple studies suggesting that DNA ejection occurs through the capsid’s 5-fold axis.^40,41^ Another factor may be the energetic cost to compress DNA inside the capsid. We estimate the 4.7 kb ssDNA in this work to have a radius of gyration of approximately 29 nm in solution, but the AAV capsid is only approximately 22 nm in diameter (details in the Supporting Information). The resultant energetic cost to compress DNA for viral packaging is supported by previous studies, where increasing DNA cargo size above 3 kb both decreases an AAV’s thermal melting temperature and increases transduction efficiency.^22^ However, the cost of compressing DNA is likely somewhat offset by stabilizing enthalpic interactions of DNA with the positively charged capsid interior.^16,42^

Figure 2D also plots the aggregation rate, AR, for the empty AAV. This was determined according to eq 12 and fit to the early phase aggregation (*M*_w_/*M*_0_ between 1 and 1.5) to minimize artifacts due to the scattering of large *M*_w_ species. Empty capsid aggregation rates are between 100-fold (53 °C) and 1.5-fold (38 °C) faster than the rate of full capsid DNA release, which is not explainable by interfacial or concentration effects. The empty AAV aggregation also obeys Arrhenius behavior but with two separate linear regimes at 35–44 and 44–53 °C with activation energies of 110 and 44 kcal/mol, respectively. These activation energies are much larger than those of the DNA release for full AAVs. The two separate regions are characteristic of biomacromolecule aggregation well below (35–44 °C) and adjacent to (44–53 °C) the so-called melting temperature, *T*_m_(10,25,39) (or *T* at which about half the unfolding has occurred). These two aggregation regimes suggest that thermal unfolding of quaternary structure (conformation) is a key driver of aggregation at the 44–60 °C range but that native state aggregation (colloidal interactions) may dominate at the lower temperatures. This agrees with the measured uncoating temperature (e.g., temperature for rapid release of DNA) of approximately 51 °C for the AAV in this work.

### Comparison of SLS and DLS Results

The contrasting thermal degradation of empty vs full AAV is further demonstrated using DLS and SLS. These measure fundamentally different mean properties: SLS measures *M*_w_, and DLS measures apparent *z*-average hydrodynamic diameter ⟨*d*_H_⟩_*z*,ap_. Note that DLS measures a *z*-average diffusion coefficient which overweights massive aggregates, whereas 90° SLS intensity gives a weight average that underweights massive aggregates. As such, SLS is likely an underestimate of mass, whereas DLS is an overestimate of apparent hydrodynamic diameter.

**Figure 3:**
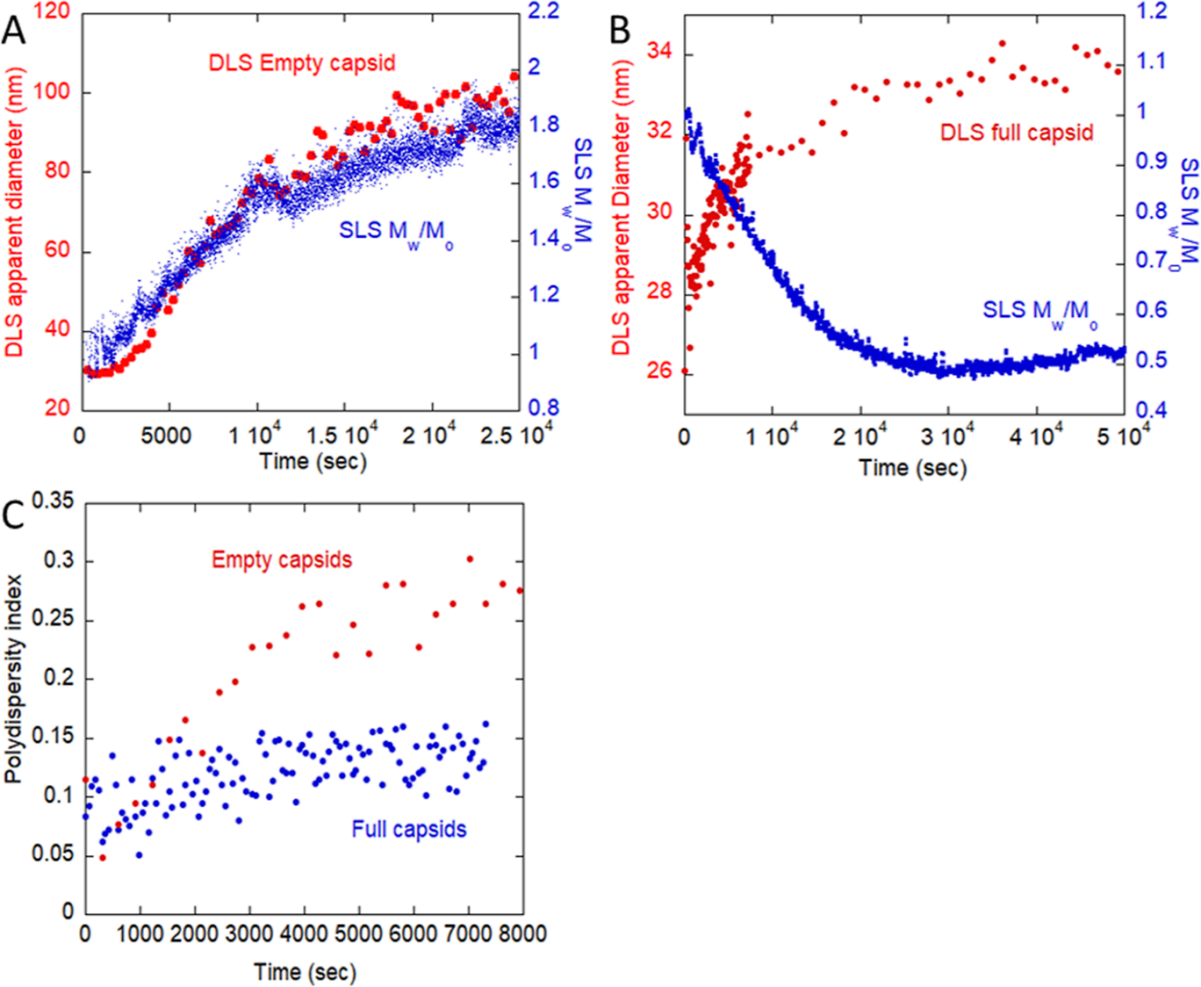
Thermal degradation of AAV samples monitored by SLS and DLS. (A) Empty AAV, ⟨*d*_H_⟩_*z*,ap_ (DLS, 44 °C), and *M*_w_ (SLS, 44 °C). (B) Full AAV, ⟨*d*_H_⟩_*z*,ap_ (DLS, 44 °C), and *M*_w_ (SLS, 44 °C). (C) Polydispersity index (PD, DLS) for empty versus full AAV.

As shown in Figure 3A, empty AAV at 44 °C exhibit qualitatively similar profiles between *M*_w_ and ⟨*d*_H_⟩_*z*,ap_ with increasing *M*_w_ (SMSLS) and ⟨*d*_H_⟩_*z*,ap_ (DLS) over time. The increase in diameter for the aggregating empty capsid is around 7-fold, which would give around 50 unfolded capsids per aggregate, on weight average, if the unfolded capsids are treated as random coils and over 300 if treated as spheres. In contrast, Figure 3B shows that for 60% full capsid, ⟨*d*_H_⟩_*z*,ap_ exhibits a small increase of 6 nm over 10 h, while *M*_w_ decreases. This is consistent with the ejection of DNA from the full capsids, as the ssDNA in solution is estimated to have a radius of gyration of at least 36 nm (below), whereas an intact AAV capsid is approximately 22 nm. Ejected DNA would expand upon ejection to a random coil somewhat larger than the AAV capsid, resulting in the observed modest Rh increase but *M*_w_ decrease. Again, there is no massive colloidal aggregation after DNA release, unlike the aggregation of the empty capsids.

A computation to estimate the radius of gyration of the ssDNA in solution is as follows: single-stranded DNA has a linear mass density of λ = 950 Da/nm.^43^ The intrinsic persistence length is *L*_p_ ∼ 1.5 nm^44^ (this ignores both electrostatic and other excluded volume effects). Thus, for DNA of *M* = 2.4 × 10^6^ Da, the contour length, *L*, is *L* = *M*/λ = 2526 nm, so that *L* ≫ *L*_p_, and the coil limit can be used to estimate the least mean-square radius of gyration. The root-mean-square radius of gyration, ⟨*S*^2^⟩^1/2^, should have a lower limit around
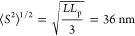
13which is larger than ⟨*S*^2^⟩^1/2^ of the intact capsid. For a solid sphere, ⟨*S*^2^⟩^1/2^ is related to the hydrodynamic diameter *d*_H_ (20 nm for the intact capsid)
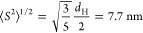
14

This supports the findings that ⟨*d*_H_⟩_*z*,ap_ increases for full capsids as they release DNA, seen in Figure 3B, since the DNA in solution has a larger effective ⟨*d*_H_⟩_*z*,ap_, while the total light scattering intensity drops as the full capsids break into subunits.

A further supporting effect for the rapid aggregation of empty but not full AAV can be seen in the DLS polydispersity index shown in Figure 3C for the empty and full capsids. Initially, both empty and full capsids are fairly uniform with low polydispersity index, PI < 0.10. As aggregation proceeds, PI quickly reaches an average value of 0.34 for the empty capsids, which is considered highly polydisperse.^45^ In contrast, the full capsids show a small increase in PI as DNA is ejected and then maintain a fairly narrow distribution of average PI approximately 0.11. This confirms that no massive, highly polydisperse aggregation of the capsids occurs.

### Degradation of Mixed Ratios of Empty and Full Capsids

**Figure 4:**
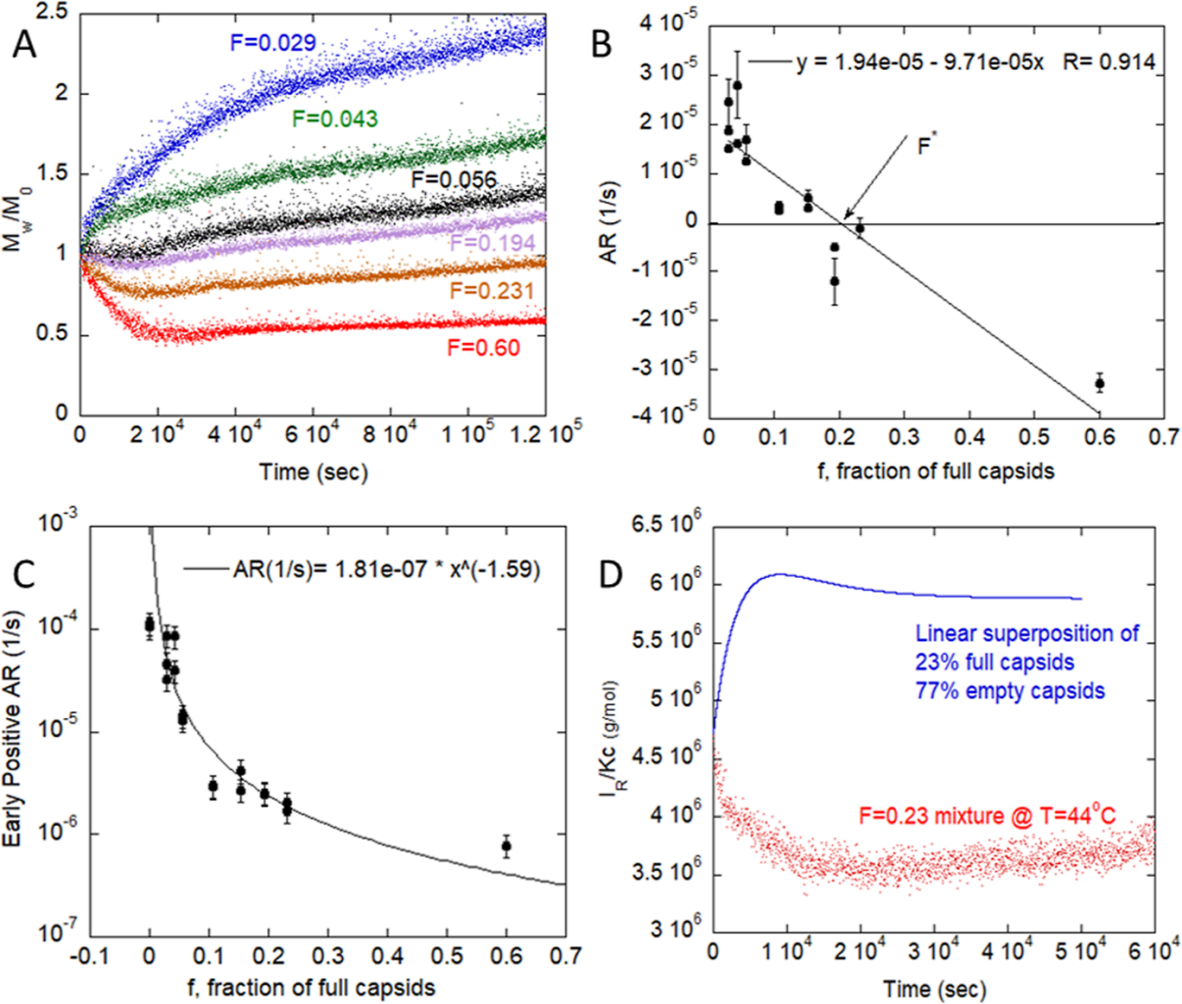
Effects of E/F ratio on AAV degradation. (A) *M*_w_/*M*_0_ of AAV at 44 °C with E/F ratio between 0.029 and 0.6. (B) Early sign crossover for AR occurs at F*. (C) Early positive aggregation rate vs % full capsids, with power law fit. (D) *I*_R_/*Kc*, the effective molar mass, for *F* = 0.23 mixture of capsids at *T* = 44 °C and the computed value (solid line) assuming linear superposition of the individual behavior of mixed and empty capsids. The disparity indicates that the empty and full capsids are not behaving independently of each other, supporting the hypothesis of DNA release affecting the degradation behavior.

The differing degradation of empty versus full AAV is notable as the encapsulated DNA is the only difference between the viral stocks. This implies several features: (a) the viral aggregation is primarily promoted by instability of the viral capsid alone and (b) the released DNA and any coreleased proteins interact to alter degradation route and lower aggregation rate of full AAV capsids. To further understand the role of encapsulated DNA in capsid degradation, we studied AAV at 44 °C and varied the fraction, *F*, of full capsids (Figure 4). SLS profiles with varied *F* but constant capsid number are shown in Figure 4A. For *F* between 0.6 and 0.056, there is an initial negative slope corresponding to DNA release, followed by slow aggregation. With decreasing *F* below 0.056, the degradation profile is overwhelmed by rapid early aggregation, with aggregation rate growing quickly as the fraction of full capsids is further decreased.

Figure 4B plots the initial slopes in *M*_w_/*M*_0_, which are negative for those with the initial drop in *M*_w_/*M*_0_, and positive for those without a drop. The point of crossover (F*) between positive and negative slopes suggests a transition in degradation mechanisms from empty-dominant to full-dominant behavior, and F* is estimated by linear interpolation to be between 0.056 and 0.20. A positive aggregation rate was computed from the slope in *M*_w_/*M*_0_ after reaching the *M*_w_/*M*_0_ minimum. This is plotted in Figure 4C with a power law fit to guide the eye. Here, AAV with *F* < 0.056 have AR between 10^–5^ and 10^–4^ s^–1^, whereas AAV with *F* > 0.056 have a much lower AR below 2 × 10^–6^ s^–1^.

As the SLS signal is the sum of all scatterers in solution, the SLS profile of a mixture of independently degrading empty and full AAV would be a linear combination of the measured empty and full degradation profiles. To demonstrate that interactions from the degradant species alter capsid degradation, Figure 4D plots the observed *M*_w_/*M*_0_ from SLS for *F* = 0.23 vs a hypothetical scattering signal of independent degradation (e.g., linear superposition of the separate empty and full capsid profiles, with *F* = 0.23). The linear superposition predicts a sharp initial rise, followed by a slow decrease in *M*_w_/*M*_0_. The measured signal is quite different, with an initial drop in *M*_w_, followed by slow aggregation. Additional evidence for strong interactions of degradant species can be seen by the crossover F*, which would be near the mass average for independent scatters (0.6) but we measure at about 0.1.

### Degradation in the Presence of Nuclease to Degrade DNA

**Figure 5:**
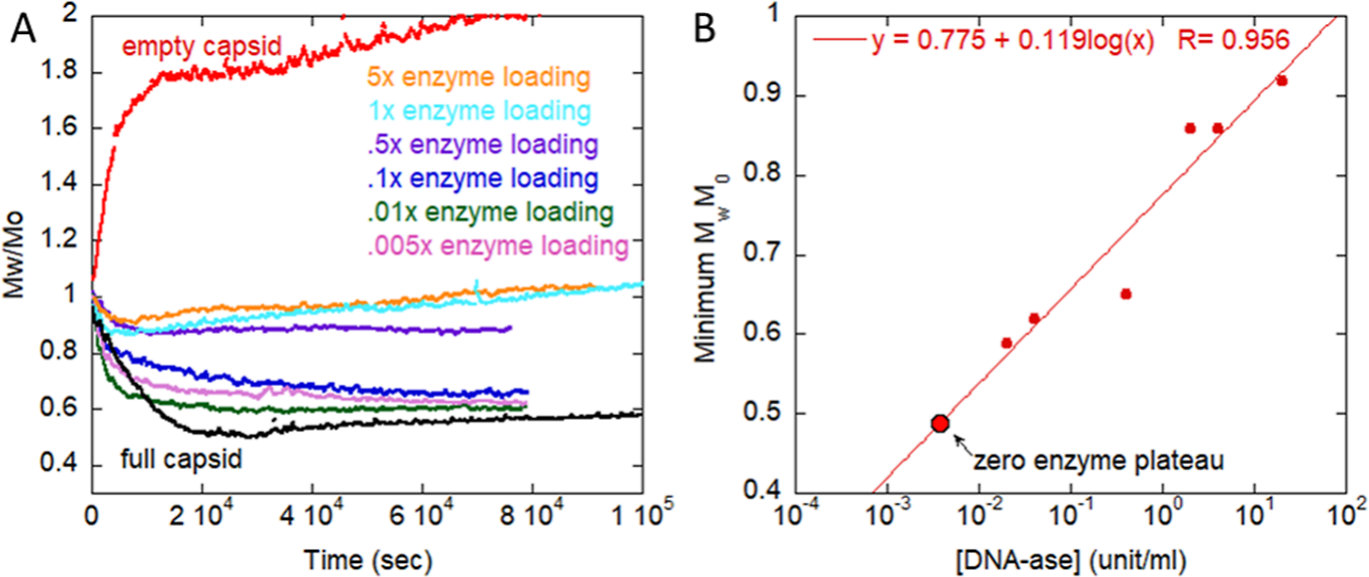
Addition of nuclease during stress of 60% full AAV reduces observed *M*_w_/*M*_0_ decrease. (A) SMLS profile of AAV during 44 °C stress, with varied nuclease concentration from 0 to 20 unit/mL to degrade DNA after release. 2 mM MgCl_2_ is added under each condition and required for nuclease activity. (B) Minimum *M*_w_/*M*_0_ reached during plateau period vs [DNAase]. A logarithmic fit is shown, as well as the value when there is no enzyme.

To further understand the role of DNA on capsid degradation, deoxyribonuclease (DNase) was added to degrade DNA not contained by an intact capsid (e.g., released during DNA ejection). The effect of enzyme concentration on 60% full AAV at 44 °C is shown in Figure 5A,B. The most prominent effect of increasing enzyme is to decrease the drop in *M*_w_/*M*_0_ during the DNA release period (Figure 5A). This follows a definite logarithmic trend, Figure 5B, where the minimum of *M*_w_/*M*_0_ is linear vs log[DNase]. One potential explanation is that DNA may be degraded as it is slowly excreted so that only a portion is fully released. However, it is unlikely that the remaining DNA would be stably contained within a disrupted capsid. Another explanation is that the released DNA is sufficiently degraded by the DNase and loses, at least partially, its ability to influence aggregation of empty and fragmented capsids. As such, the decrease in *M*_w_ from DNA ejection would be offset by the simultaneously occurring increased capsid aggregation. In the event that the DNA was released and degraded, without affecting empty capsid degradation, the minimum *M*_w_/*M*_0_ would be lower than in the no-DNase case since the only massive scatterers remaining would be the capsids and/or their protein fragments rather than these scatterers plus released, intact DNA, as in the above cases without DNase.

Another notable feature with increasing [DNase] is a slight but noticeable increase in aggregation following DNA ejection. This increase in aggregation does not fully recover the magnitude and rate seen in empty AAV. However, it is still reminiscent of the similar increase when increasing the percentage of empty capsids in an empty/full mixture (Figure 4A). Most importantly, because the DNase only degrades extra-viral DNA and does not directly influence capsid proteins, this confirms that excreted, high-molecular weight DNA is a critical component in influencing the degradation pathway of the empty vs full capsids demonstrated in Figures 2 and 3.

### Aggregation to Form μm-Sized Particulates

Another feature of the SLS degradation profiles is the presence of large data point scatter in the scattering intensity. This “noise” is caused by the diffusion of large scatterers, greater than about 150 nm diameter, in and out of the scattering volume. The contribution is strongly dependent on size: particles above approximately 120 nm diameter have about a 10% underestimate of molar mass at 90° detection, an error that grows rapidly with further increase in size. The large aggregates causing the noisy band thus have masses far in excess of the measured *M*_w_/*M*_0_ on the order of only 2 (details of how single-angle detection begins to rapidly underestimate mass, due to Mie scattering effects, are given in the Supporting Information).

Data point scatter, and thus particulate presence, is observed throughout thermal degradation studies of both empty and full AAV. Particulates are especially prominent in the aggregation phase of all samples, with fluctuation amplitudes generally larger for samples with a high fraction of empty capsids. This increased fluctuation amplitude for empty capsids may arise from both increased particulate numbers and/or a bias toward the 100–500 nm size range. Both explanations are supported by the increased PD for stressed empty vs full AAV from DLS (Figure 3C).

**Figure 6:**
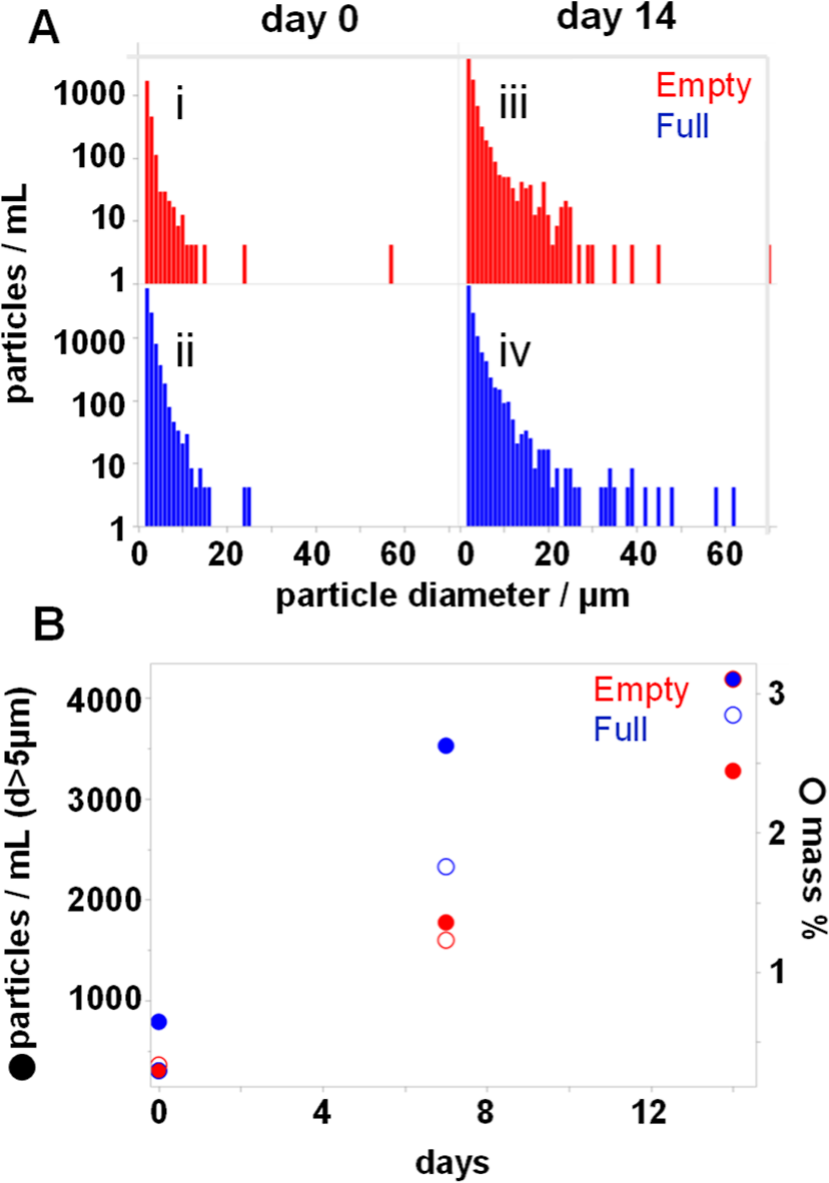
Formation of micron-size subvisible particulates in empty and full samples during 35 °C incubation. (A) Particle size distributions from backgrounded membrane imaging across 2 weeks of exposure. (B) Number of subvisible particles with diameter >5 μm and estimated fraction of AAV mass present as subvisible particles assuming 900 g/m^3^ protein density and a uniform 1 μm thickness on the membrane.

Backgrounded membrane imaging (BMI) was used to further interrogate the formation of particulates that were >1 μm in size. BMI retains particles from a sample onto a filter membrane and determines their size, shape, and intensity using automated microscopy and image analysis (example images in Figure S2). Figure 6A shows example particle size histograms from empty and full AAV during 35 °C stress at equivalent capsid concentration by SEC-MALS. As typically expected, the largest particle populations are in the 1–2 μm size bin (smallest measurable by this technique). The particle populations decrease exponentially with increasing size into the ∼20–40 μm range.

The overall area of particles was used to estimate net particle amounts before and after thermal stress (Figure 6B). This estimation of 3D spatial features from a 2D reporter is somewhat reasonable, as internal evidence suggests that the proteinaceous particles flatten on the membrane during particle isolation (further discussion in the Supporting Information). The mass of identified particulates was estimated from the total particle area (Figure 6B) by assuming a protein density of 900 g/m^3^ and a uniform 1 μm particle thickness. This is expected to overpredict total particle mass, as the low scattering intensity during side illumination of even 25+ μm particles is indicative of submicrometer thicknesses after membrane filtration to remove liquid (further discussion in the Supporting Information). The identified particles account for less than 3% of the overall AAV mass for both empty and full samples. Thus, particulates are an insignificant mass fraction during the events observed by light scattering and are not expected to significantly bias the observed scattering profiles and interpretations.

## Discussion

The most notable finding of this study is the contrasting degradation of empty (fast colloidal aggregation) and full (genome ejection, followed by slow aggregation) AAV in physiological solution and moderate temperature. One explanation may be that encapsulated DNA causes colloidal or structural stability differences between the full and empty AAV particles. DNA has been observed to effect capsid structure.^46,47^ DNA also contributes significant negative charge, potentially increasing the electrostatic stabilization at the pH 7.2 of this work (*pI* values for empty and full are between 6.0 and 6.5). These effects may all reduce initial aggregation propensity of full vs empty AAV, which was observed. However, these explanations would presumably lead to rapid capsid aggregation after DNA release, which was not observed.

Another explanation may be differing properties of empty vs full AAV. Both are formed due to stochastic assembly of AAV proteins and transgene during bioreactor production. As such, differences in polydispersity of empty vs full capsids are not just expected but may create different net properties. This may result in differences in the *vp* ratio, capsid integrity, or other features that would impact intraparticle interactions and thus capsid stability. Indeed, differences between empty and full AAV have been reported at both ensemble and single-particle levels.^48,49^ However, while this may explain some qualitative differences between empty and full AAV aggregation, it does not explain the increased aggregation of full AAV after nuclease addition or how mixtures of empty and full AAV do not have superimposable degradation profiles. Instead, both of these behaviors imply a clear and important role of the degradation byproducts and especially the ejected DNA in influencing the capsid degradation pathway.

### Aggregation Reduction Is Not Due to Overlapping DNA Chains

One conjecture for the reduction in aggregation due to DNA ejection is that it might form a network of overlapping chains, slowing down the diffusion of viral capsids. A straightforward computation shows that this is highly unlikely. Flory’s equation for relating *M* and the mean-square radius of gyration, ⟨*S*^2^⟩, can be used to compute intrinsic viscosity [η] of an ideal coil via
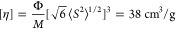
15where Φ = 2.56 × 10^23^ (mol^–1^). A useful approximation is that the overlap concentration, c*, for a polymer solution occurs at 1/[η]. So, for this particular DNA
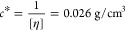
16

The concentration of DNA in a solution of 1.5 × 10^–5^ g/cm^3^ of full capsids is given approximately as follows. The full capsid *M*_w_ was measured as 8.2 × 10^6^ g/mol, so that DNA represents about 29% of the total mass, so that the DNA concentration in solution, when fully released, is *c*_DNA_ = 5.3 × 10^–7^ g/cm^3^, which is nearly 50,000× below c*. Hence, DNA is not expected to form an overlapping network at the concentration used, so viral aggregation is not due to being impeded by such a solution-wide network.

### Conjectures on the Observed Degradation Behavior

The failure of superposition of empty and full degradation to mimic degradation profiles of mixtures suggests strong interactions among components that affect degradation mechanism. Because the addition of nuclease largely recovers the degradation profile of empty AAV, intact DNA and associated components are a critical component in these interactions. Together, these results suggest a key role for intact, excreted DNA in reducing the rate of early phase capsid aggregation. However, there is no obvious explanation of how or why the material released by the capsids causes the large reduction in the aggregation rate and extent. Estimates of the DNA’s solution properties above demonstrate that it is far below the overlap concentration in this work, so that any reduction in capsid aggregation is not due to viscoelastic effects or the formation of a network of released DNA in solution.

One conjecture for the reduced aggregation of full AAV is stabilizing interactions between excreted DNA and associated proteins with AAV capsids. Significant hydrophobic area is present between capsid protein contacts, which would be exposed in capsid fragments, causing rapid aggregation. Interactions with DNA may stabilize such compromised capsids as DNA–capsid fragment complexes. Such complexes are likely as the packaged DNA has both specific interactions with packaged AAV proteins and nonspecific interactions with AAV capsid proteins.^16,42^ Such DNA–fragment complexes have been observed in single-molecule studies of AAV under mild to moderate thermal stress.^9,22^ However, this does not fully explain the large, initial drop in *M*_w_ observed by SLS for full AAV because if the DNA stays significantly associated with the parent capsid, then there would be little or no drop in the SLS intensity. Furthermore, if the DNA remained localized, it does not explain how adding a small fraction of full DNA-releasing capsids can stabilize a much larger population of aggregation-prone empty capsids.

Another conjecture as to the role of DNA may lie in the steric stabilization of elastic collisions. Since the DNA, once released from the capsid seems to be completely detached (LS decreases), capsids, whether empty or full, may make elastic collisions with the DNA during diffusion-controlled encounters and just “bounce off”. The concentration of capsids is low in these experiments and particles (sum of both empty and full capsids) at ∼2.6 × 10^12^ particles/cm^3^ have an approximate mean free path of 7.2 × 10^–5^ cm, or 720 nm. A capsid particle of *D*_H_ = 20 nm has a self-diffusion coefficient of *D*_0_ = 2.5 × 10^–7^ cm^2^/s in water at *T* = 25 °C. The average time, ⟨*t*⟩, for the particle to diffuse a distance *L* is given by
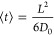
17so the 20 nm capsid would have ⟨*t*⟩ ∼ 3.5 × 10^–3^s to diffuse over *L* = 720 nm and a diffusing capsid could reach *L* with a frequency of *f* = 290 times per second. A rough estimate of the number of times there would be a collision with another capsid during these 290 diffusive segments of length *L* can be made by considering a simple lattice picture, where there would be, on average, six capsids at distance *L*, 2 for each of three independent spatial axes. The number of collisions with capsids per second, *N*, would then be
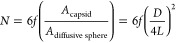
18where *A*_capsid_ = π*D*^2^/4 is the cross-sectional area of a capsid of diameter *D* and *A*_diffusive sphere_ = 4π*L*^2^ is the surface area of the diffusive sphere of radius *L*. Using *D* = 20 nm, from the capsid hydrodynamic diameter and *f* = 290 s^–1^ gives *N* ∼ 0.084 diffusion-controlled collisions/s of a given capsid with other capsids.

As an example, the data from Figure 4A, for empty capsids at 44 °C, yield an AR of 0.00011 s^–1^. A convenient interpretation of AR is that its reciprocal is the time to the average dimerization of capsids, *t*_d_. At 44 °C, this time is about *t*_d_ ∼ 9100 s. Using *N* ∼ 0.084 gives an estimate of ∼760 collisions needed to cause a single pair of empty capsids to stick together. This suggests that the chances of two colliding, (partially) unfolded capsids sticking together in any single encounter is very small and may require fairly strict geometrical orientations of the two capsids for sticking to occur. The Arrhenius data of Figure 2D can be used to estimate the number of collisions needed to cause a single pair of capsids to stick together vs *T*.

In this conjecture, the role of DNA in slowing empty capsid aggregation may stem from its considerably greater size and hence target area than the capsids, and a large coil molecule of DNA interposed between two capsids on a path to collision would elastically bounce one of the capsids off and prevent the collision. This would slow the aggregation of empty capsids down, not stop aggregation altogether and, as seen in Figure 4A,C, increasing DNA concentration, by increasing the fraction of full capsids in the mixes of empty/full capsids, dramatically lowers the aggregation rate, but does not stop it.

As shown above, the root-mean-square radius of gyration, ⟨*S*^2^⟩^1/2^_,DNA_, for single-stranded DNA of 2.4 × 10^6^ g/mol is ⟨*S*^2^⟩^1/2^_,DNA_, in the coil limit, with a linear mass density of 950 g/mol nm, and a persistence length of 1.5 nm. The equivalent root-mean-square radius of gyration of a *D* = 20 nm capsid is *R*_g,capsid_ ∼ 7.7 nm. At equal numbers of capsids and released DNA, the probability of an elastic collision with DNA rather than a collision with a capsid is (⟨*S*^2^⟩^1/2^_,DNA_/⟨*S*^2^⟩^1/2^_,capsid_)^2^ ∼ 22. At this level of released DNA, a given capsid is 22 times more likely to make an elastic collision with a free DNA molecule than with another capsid. As the fraction of empty capsids increases in the mix, the frequency of capsid/free DNA collisions decreases.

Naturally, it would be interesting to see if exogenous DNA added to the solution inhibited aggregation, similar to the DNA released from the capsids. Unfortunately, no exogenous DNA was available to attempt this.

## Conclusions

This work studied the degradation of AAV under moderate thermal stress (30–53 °C) and in physiological solution (pH 7.2, 180 mM NaCl). Light scattering measurements at high temporal resolution, supported by orthogonal characterization methods, show that empty AAV exhibited very rapid, colloidal-type aggregation with activation energies of 110 kcal/mol (35–44 °C) and 44 kcal/mol (44–60 °C). In contrast, full AAV exhibited the release of DNA cargo with an activation energy of 25 kcal/mol, followed by very slow aggregation. Degradation profiles of E/F mixtures were not superimposable from combinations of empty and full degradation profiles, indicating that released full capsid content influences the aggregation of the empty capsids.

Experiments probing the E/F ratio and digestion of released DNA showed that DNA is a major component in influencing the capsid degradation pathway. This suggests that colloidal and/or structural instability from the capsid proteins is inducing capsid aggregation and that the ejected DNA alters the degradation path of capsids and capsid fragments. While we have not as yet undertaken an experimental strategy to explore the detailed mechanism and pathways, it may include some combination of attractive interactions that stabilize otherwise aggregation-prone capsid fragments, steric stabilization of intact capsids through both elastic and inelastic collision effects, and fundamental differences in empty vs full AAV integrity.

Together, results have key implications toward the storage of AAV pharmaceuticals. The differing degradation of empty versus full AAV suggests that empty content and DNA cargo will influence degradant species and degradant formation rates during drug product storage. AAV degradation and storage lifetime may thus be dependent on not just capsid serotype, storage temperature, and storage solution matrix but also DNA size and abundance of product variants such as empty capsids.

While the ensemble-average measurements in this work are capable of high temporal resolution, they do not provide insight into the subpopulations formed during AAV degradation. Additional studies capable of resolving the various populations are thus required to better understand the degradation pathways of the AAV variants and components. Because the insights of this work are limited to physiological solutions for one AAV serotype, further characterization under a variety of serotypes, pH, ionic strength, and other additives is also required to understand the detailed interactions and their impact on AAV degradation pathways.

## Supporting Information Available

Measurement of capsid titer during thermal degradation and particulate assessment using backgrounded membrane imaging (PDF)
