# Supplementary material for: DNA Released by Adeno-Associated Virus Strongly Alters Capsid Aggregation Kinetics in a Physiological Solution
Source: Biomacromolecules. 2024 Apr 29;25(5):2890–901. doi: 10.1021/acs.biomac.4c00027 (PMC11094734; doi:10.1021/acs.biomac.4c00027)
Supplement: Supplementary file 1 — bm4c00027_si_001.pdf [file bm4c00027_si_001.pdf]

Supplemental Information

Curtis W. Jarand,[1] *Karen Baker,* [2] *Matthew Petroff,*[2] *Mi Jin,* [2] *Wayne F. Reed*[1]*

[1] Tulane University, Department of Physics. New Orleans, LA 70118

[2] Spark Therapeutics, Downstream and Drug Product Process Development. Philadelphia, PA 19143

Measurement of capsid titer during thermal degradation

Full AAV vectors were independently stressed and assayed for comparison to the solution-average measurements by light scattering. 60% full AAV were incubated at between 30-60°C, sampled, and quenched to 4°C before further characterization. Vector integrity and DNA encapsulation were assayed by two methods: (a) anion exchange analytical chromatography (HP-AEX) interpreted changes in full capsid titer from relative changes in the area of the full peak (absorbance at 280 nm); and (b) quantitative polymerase chain reaction (qPCR) measure genome titer, with encapsulated genome content measured by pre-treating samples with nuclease to degrade unprotected genome before concentration measurement, and total genome titer measured without pretreatment with nuclease.

Changes in AAV titer during degradation are shown in Figure S1 as monitored by HP-AEX and qPCR over a range of temperatures. As monitored by HP-AEX, viral titer decreases to zero between 5-35 hours (for 60-35°C). As monitored by qPCR, samples with DNase pre-treatment (measure encapsulated titer) decrease to approximately 10-30% over 8-20 hours (for 60-40°C), while measurements without DNase pre-treatment (measuring total solution titer) show no net change. Some difference in plateau titer value is expected between the assays as HP-AEX measures both capsid and DNA integrity, whereas qPCR measures protected DNA titer. The increased qPCR plateau thus suggests that some DNA are protected by capsid degradants are fragments. More importantly, these time scales to reach the plateaus align with the Mw decrease observed in SLS (Figure 2), showing that the Mw decrease is cause by some DNA ejection and associated capsid disruption.

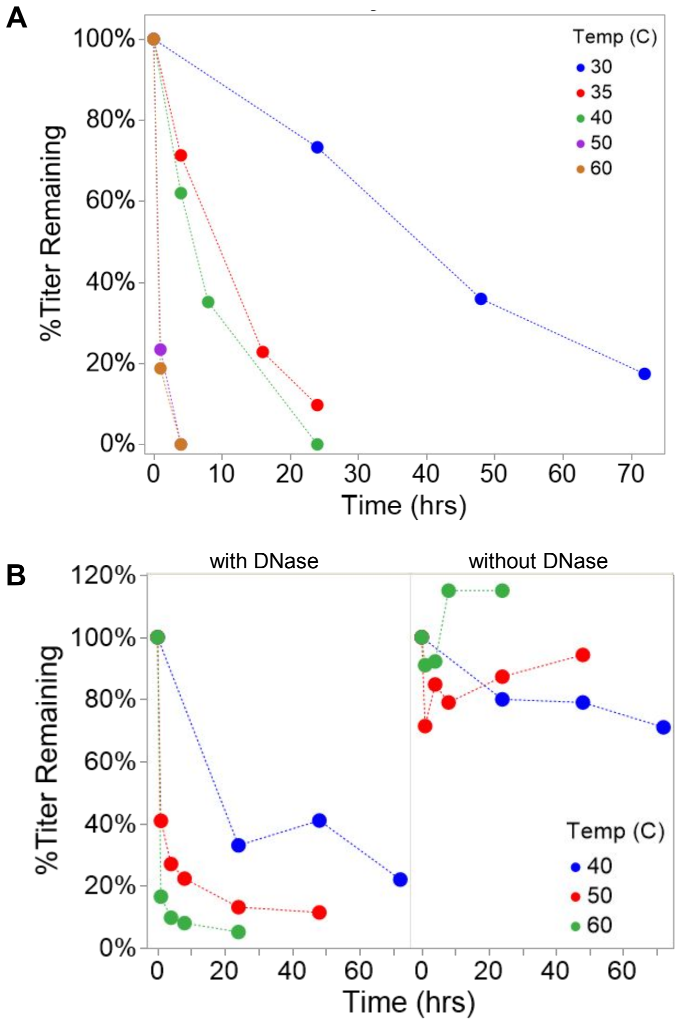

Figure S1. AAV degradation kinetics monitored by HPLC and qPCR. (A) Titer loss kinetics monitored by HP-AEX. % loss was estimated from the change in full AAV peak area relative to a control. (B) Titer loss kinetics monitored by qPCR. Samples are measured with and without DNase pretreatment to measure DNA titers of AAV within capsids (e.g. viral genome titer) and total titer in solution, respectively. The % remaining titer is estimated relative to the titer of an unstressed control.

Particulate assessment

Subvisible particulates were detected using Backgrounded Membrane Imaging (BMI) using the Aura from Halo Labs, and imaged as described in the main and following standard procedures from Halo Labs. Example images are shown in Figure S2A for a full AAV after moderate thermal stress, with brightfield (i); side-illumination, a darkfield analog (ii); Thioflavin-T fluorescence to detect stain for protein (iii); and an overlay of imaging modes (iv). Detected particles are replotted in Fig. S3B as spheres of equivalent area for ease of visualization, with colors representing particles of radius 1-5um (blue), 5-10um (red), 10-25um (green), and 25+ um (purple). The particle size distribution is plotted in Figure S2C.

One notable feature of the subvisible particle images is the low intensity of proteinaceous particles under side-illumination. This was consistent across subvisible particles formed from empty, full, and protein control feedstocks, and particle sizes (great than 99% between 1-25 μm^2 detected for all samples). The protein content of these particles was confirmed by the Thioflavin-T fluorescent stain. Side-illumination intensity is caused by scattering of particles of significant height offset from the membrane, and significant intensity was observed for even 2 μm polystyrene colloids. As such, it implies that the proteinaceous particulates flatten on the membrane after filtration, with post-filtration thicknesses of below 2 μm. We used this to estimate the maximum overall mass of subvisible particulates in a sample using the total detected particle area, a protein density of 1.35 g/mL, and an average particle thickness of 2 μm. These numbers are expected to be worst-case estimates via overestimation of the particle thickness.

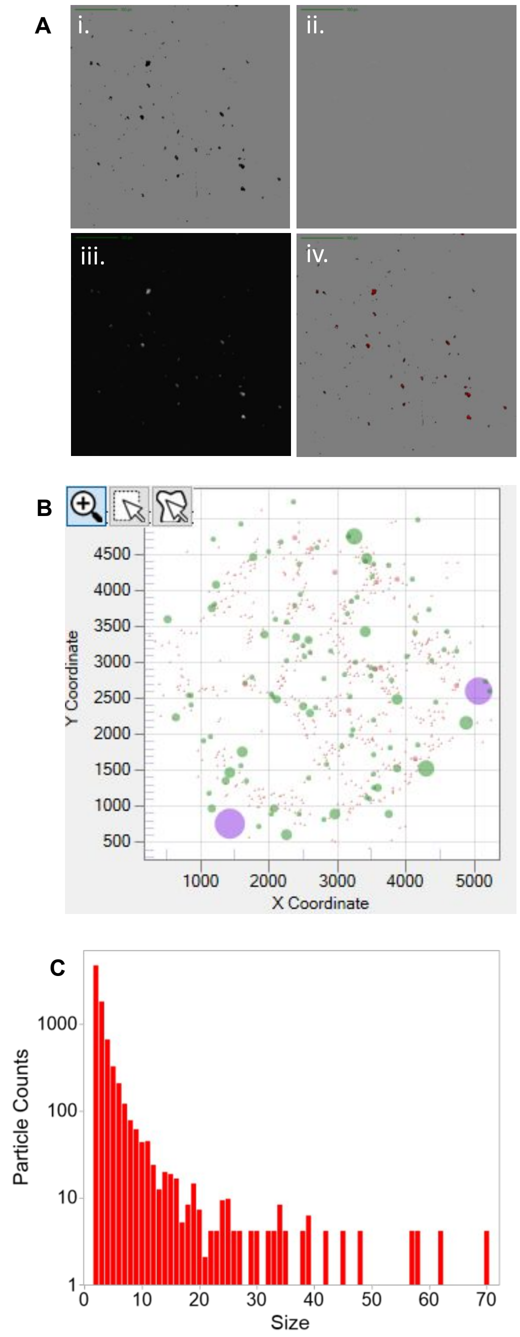

Figure S2. Example subvisible particulate detection by backgrounded membrane imaging (BMI). In all panels, 98% full AAV . **A. Imaging Modes and Data Processing:** brightfield (i), side-illumination (eg darkfield, ii), THT-fluorescence (iii), and overlay (iv). All images in same magnification. **(B)** Reconstructed schematic of identified particles plotted as spheres. Each node is centered at a particle's X-Y orientation, with area proportional to the particle area. **(C) Size distribution** of particles detected in panel A.
